# Important fossil source contribution to brown carbon in Beijing during winter

**DOI:** 10.1038/srep43182

**Published:** 2017-03-07

**Authors:** Caiqing Yan, Mei Zheng, Carme Bosch, August Andersson, Yury Desyaterik, Amy P. Sullivan, Jeffrey L. Collett, Bin Zhao, Shuxiao Wang, Kebin He, Örjan Gustafsson

**Affiliations:** 1 SKL-ESPC and BIC-ESAT, College of Environmental Sciences and Engineering, Peking University, Beijing 100871, China; 2 Department of Environmental Science and Analytical Chemistry (ACES) and the Bolin Centre for Climate Research, Stockholm University, Stockholm 10691, Sweden; 3 Department of Atmospheric Science, Colorado State University, Fort Collins, Colorado 80523, USA; 4 State Key Joint Laboratory of Environment Simulation and Pollution Control, School of Environment, Tsinghua University, Beijing 100084, China

## Abstract

Organic aerosol (OA) constitutes a substantial fraction of fine particles and affects both human health and climate. It is becoming clear that OA absorbs light substantially (hence termed Brown Carbon, BrC), adding uncertainties to global aerosol radiative forcing estimations. The few current radiative-transfer and chemical-transport models that include BrC primarily consider sources from biogenic and biomass combustion. However, radiocarbon fingerprinting here clearly indicates that light-absorbing organic carbon in winter Beijing, the capital of China, is mainly due to fossil sources, which contribute the largest part to organic carbon (OC, 67 ± 3%) and its sub-constituents (water-soluble OC, WSOC: 54 ± 4%, and water-insoluble OC, WIOC: 73 ± 3%). The dual-isotope (Δ^14^C/δ^13^C) signatures, organic molecular tracers and Beijing-tailored emission inventory identify that this fossil source is primarily from coal combustion activities in winter, especially from the residential sector. Source testing on Chinese residential coal combustion provides direct evidence that intensive coal combustion could contribute to increased light-absorptivity of ambient BrC in Beijing winter. Coal combustion is an important source to BrC in regions such as northern China, especially during the winter season. Future modeling of OA radiative forcing should consider the importance of both biomass and fossil sources.

Organic aerosol (OA) is a major component of fine and ultrafine particles with significant impacts on air quality, human health, and climate^1^,^2^. In particular, radiative forcing of OA, a critical component of the aerosol radiation budget, has drawn increasing attention as many studies have indicated that some OA can absorb light (defined as Brown Carbon, BrC)^3^,^4^ and be responsible for ~20% to >50% of the total aerosol light-absorption at UV wavelengths^5^,^6^,^7^,^8^. Brown carbon contributes to both direct and semi-indirect effects of aerosol radiative forcing, yet uncertainties abound. There are a growing number of observations of light absorptive BrC^9^, and the need to include OA absorption in radiative transfer and chemical transport models is clear. Up to now, only a few studies have been conducted on radiative forcing of BrC, primarily associated with the still poor understanding of tropospheric BrC burden as well as its optical properties^5^,^10^,^11^,^12^. By constraining BrC absorptivity in a global carbonaceous aerosol emission inventory and using fuel-type-based refractive indices derived from recent laboratory and field observations, Lu *et al*.^13^ indicated primary organic aerosol (POA) could warm the atmosphere significantly and lead to ~27% reduction of the net global average POA cooling effect under the non-absorbing assumption, but secondary formation and other atmospheric processing were not included in the estimates. Better parameterization of sources and source-specific OA light-absorbing properties, as well as atmospheric processing from source emission to ambient aerosol, are critical and urgently needed to better understand the climate impacts of OA. However, knowledge on emissions and sources of global BrC and impacts of atmospheric processing is still limited.

As for sources of BrC, although laboratory test-burn studies have examined optical properties of OA from different sources and demonstrated OA absorptivity can be highly source dependent^4^,^14^,^15^,^16^, current light-absorbing OA studies have mainly focused on biomass/biofuel combustion both in field observations^17^,^18^,^19^ and in smog chamber-based laboratory studies^14^,^16^,^20^. Only a few studies have suggested that fossil sources such as coal combustion^8^,^21^,^22^ or non-biomass burning anthropogenic emissions^23^,^24^ could contribute to BrC. The few radiative-transfer model and chemical-transport model studies that so far have at all considered BrC effects have assumed that biomass or biofuel burning are the main or sole BrC source without any regional and seasonal difference in parameterization^5^,^10^,^11^. In addition, current field studies mostly focus on optical property and source of water-soluble BrC^19^,^25^,^26^,^27^,^28^,^29^,^30^,^31^,^32^, seldom examining water-insoluble BrC.

Studies on sources and source-specific properties of ambient BrC are particularly rare in key regions such as China, which is associated with high carbonaceous aerosol emissions^33^, especially from anthropogenic sources including POA and secondary organic aerosol (SOA) contributions^34^,^35^. Current field studies attribute BrC in megacity Beijing and polluted air outflow from northern China to high biomass burning emissions and photochemical SOA formed from anthropogenic precursors^25^,^26^, or estimate its source as residential biofuels or coal combustion^36^. Higher mass absorption coefficient for air masses from Northern China observed in Gosan was suggested to be due to higher fraction fossil source contributions^37^. However, the relationship between OA sources (especially anthropogenic sources such as Chinese-characteristic coal combustion) and light-absorbing properties needs to be investigated in depth, and optical as well as chemical properties should be measured directly.

Recent advances in isotope measurements allow quantitative source fingerprinting. Radiocarbon (^14^C) analysis provides powerful and quantitative information to differentiate relative contributions of fossil fuel (e.g., oil, petroleum, natural gas and coal) versus biomass burning/biogenic emission (BB/BG) sources to carbonaceous aerosol^28^,^29^,^38^,^39^. While ^14^C fingerprinting has been applied to total carbon^40^,^41^, elemental carbon (EC) and organic carbon (OC) fractions^38^,^42^, there are also some promising applications to specific subsets of OA such as water-soluble organic carbon (WSOC)^28^,^29^,^30^,^37^,^43^, water-insoluble organic carbon (WIOC)^28^,^44^,^45^, and polycyclic aromatic hydrocarbons (PAHs, 46), making it possible for combined investigation on sources and optical properties of light-absorbing carbonaceous aerosol^29^,^30^,^37^. Additionally, analysis of stable carbon isotope (^13^C) could provide further information regarding sources and atmospheric processing^29^,^30^.

Beijing is a city representing areas with high anthropogenic carbonaceous aerosol emissions (Fig. S1). Better understanding on the fossil versus BB/BG source contributions, and associated light absorption by carbonaceous aerosol is essential for formulating emission-based control strategies and constraining uncertainties in current aerosol radiative forcing estimates. Here, we investigated light-absorbing properties of both water-soluble and water in-soluble BrC in the atmosphere and from Chinese characteristic source emissions, and distinguished fossil and BB/BG source contributions based on carbon isotope measurement.

## Results and Discussion

### Seasonal variation of OA component and its light absorption

Atmospheric OA levels in Beijing showed a clear seasonal variation. During two field campaigns conducted in the summer and winter of 2013 in Beijing, 24-h average atmospheric OC loading and its two sub-constituents (WSOC and WIOC, calculated as the difference between OC and WSOC, with propagated uncertainty of 9%) were a factor of 3, 1.5 and 5 times greater in winter than summer, respectively. WIOC, accounting for a higher proportion of total OC in winter (WIOC/OC: 69 ± 5%, with propagated uncertainty of 12%) compared to summer (40 ± 6%), was responsible for ~85% of the increased OC mass in winter. Stronger correlations of both WIOC and WSOC with EC in winter (r^2^ = 0.85 and r^2^ = 0.75, respectively) than summer (r^2^ = 0.22 and r^2^ = 0.01, respectively), suggested that the higher wintertime WIOC and WSOC concentrations in Beijing were mainly related to combustion sources.

Water and methanol extracts of wintertime fine particle (PM_2.5_) samples showed higher light-absorbance compared to summer, with stronger light-absorption by methanol extracts than water extracts at all wavelengths, especially at shorter wavelength (<400 nm) (Fig. S2). This might be associated with extra light-absorbing components extracted by methanol, such as large molecular weight PAHs and quinones, from either fossil-fuel and/or biomass burning^14^,^31^. Here, water-extracted light-absorbing OC is defined as water-soluble brown carbon (WS-BrC). Since most OC can be extracted by methanol^14^, it is here assumed that the difference in light-absorption by methanol and water extracted BrC is attributed to WIOC (defined as water-insoluble BrC or WIS-BrC). It is noted that the mass absorption efficiency (MAE) of WIS-BrC at 365 nm (MAE_365_) calculated here acts as a first-order estimate due to the actual extraction efficiency of OC by methanol lower than 100% and the coexistence of non-light-absorbing organic compounds. MAE_365_ values of wintertime WIOC and WSOC were about 1.3- (*p* > 0.05) and 2.5-fold (*p* < 0.05) higher than that of summertime, respectively (Table 1). Light-absorptivity of WIOC was on average 2 times higher than for WSOC in summer (*p* < 0.05), while in winter the MAE values for WIOC and WSOC were nearly identical (*p* > 0.05). The difference in MAE_365_ of WIOC and WSOC during winter and summer might be ascribed to chemical compositions of OA from different source emissions or due to differences in atmospheric transformation after emissions. By contrast, the differences between Absorption Ångström Exponents (AAE) over the range of 340–400 nm of WSOC and WIOC and their seasonal variations were not significant (Table 1).

### Fossil versus biomass/biogenic source of OA components

**Figure 1:**
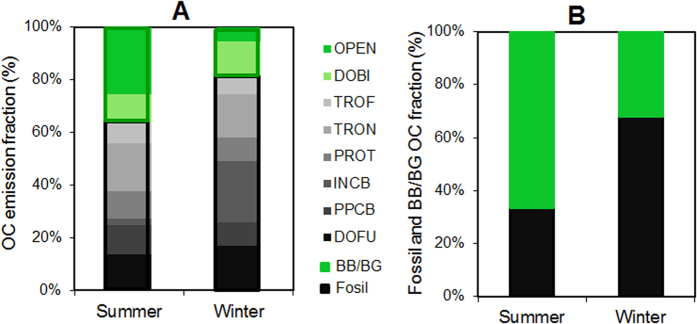
Emission sources of OC aerosol in Beijing as predicted by “bottom-up” emission inventory (EI; primary OC) and “top-down” isotope-based source apportionment of ambient atmospheric OC. Panel A: Predictions of source contributions from EI for primary OC aerosol in Beijing. Bars filled with green color denote biomass/biogenic source emissions; Bars with black-grey color denote fossil source emissions. Fossil source includes “TROF” (off-road transport), “TRON” (on-road transport), “PROT” (other industry processing), “INCB” (industry combustion), “PPCB” (power plants), “DOFU” (domestic fossil fuel combustion), and biomass/biogenic source includes “DOBI” (domestic biomass combustion) and “OPEN” (open biomass burning). Panel B: ^14^C-observation-based source apportionment of ambient organic aerosol in Beijing between fossil *vs.* biomass/biogenic sources.

Radiocarbon measurements, a powerful tool to discern between fossil fuel versus BB/BG sources of different organic components, showed clear and significant contribution of fossil source in Beijing winter. The ^14^C signatures in all type of OC fractions (i.e., OC, WSOC and WIOC) exhibited higher fossil fraction contributions (>50%) in winter compared to summer (~30%). The fossil source contributions to OC and WSOC in the current study (about half) were consistent with some limited previous results for the outflow from East Asia (Table S1), but higher than what have been reported in winter of 2011 in Beijing (OC: 45 ± 5%, and WSOC: 43 ± 3%)^46^, and that in Gosan (WSOC: 30–50%)^37^, in South Asian outflow such as in Maldives (OC: 31 ± 5%; WSOC: 14 ± 5%)^29^, and in Delhi, India (OC: 46 ± 8%; WSOC: 21 ± 4%)^30^. Compared to the Beijing-tailored emission inventory (Fig. 1), the relative contributions of fossil and BB/BG sources to wintertime OC in Beijing from the top-down radiocarbon-based observations agreed reasonably well with that based on predictions from bottom-up EI (see “*Emission Inventory*” and “*Bottom-up Emission Inventory versus Top-down*^*14*^*C Measurement*” in the supplementary).

**Figure 2:**
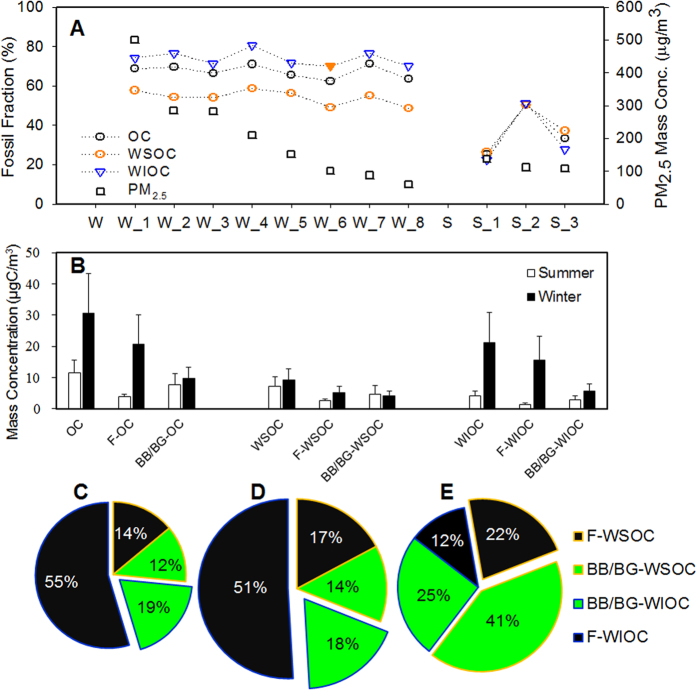
Fossil and biomass/biogenic source distributions of OC and its sub-constituents in Beijing. Panel A: Variations of fossil fractions in OC and its sub-constituents with PM_2.5_ mass concentrations during the winter (W) and summer (S) campaigns. Panel B: Averaged absolute concentrations of fossil and non-fossil OC, WSOC and WIOC. Panel C–E: Fossil (F, gray filled) and biomass burning/biogenic source (BB/BG, white filled) contributions to different OC constituents during days with PM_2.5_ <100 μg m^−3^ in winter (Panel C), and days with PM_2.5_ >100 μg m^−3^ in winter (Panel D) and summer (Panel E). Sectors with orange line denote WIOC and blue line for WSOC. The size of each pie is correlated with the PM_2.5_ mass concentration.

WIOC had the highest fossil fuel contribution (70–77%) in winter, followed by OC (62–71%) and WSOC (49–59%), with lower yet still high fossil fractions in summer for WSOC (26–50%), OC (25–51%), and WIOC (23–51%) (Table 1). It should be noted that, the fossil fraction of wintertime OC, WSOC, and WIOC were independent of PM_2.5_ mass concentration (Fig. 2A), suggesting a stable and predominant contribution of fossil fuel combustion to wintertime OA in Beijing. Compared to summer, average concentration of fossil OC, WSOC and WIOC increased by 5, 2, and 12 times in winter, respectively (Fig. 2B). The different degree of increases of OC and its two sub-constituents implied the distinct source contributions in the two seasons, beyond unfavorable meteorological conditions (e.g., low wind speeds or low mixing layer heights)^47^. The 5-fold increase of wintertime fossil OC could be primarily attributed to the increased primary emissions such as ubiquitous coal combustion for residential cooking and heating and coal-fired industry in winter (Fig. 1 and Fig. S3). The striking increase of fossil WIOC (~12 times), occupying 70% and 83% of the increased OC and WIOC mass in winter, respectively, played a predominant role in the increase of wintertime fossil OC. Subsequently, a 2-fold increase of fossil WSOC in winter indicated fossil source could also contribute to WSOC loading. This is further supported by coal combustion source testing in this study, demonstrating 16 ± 7% of OC emitted from residential coal combustions is water-soluble. In contrast, BB/BG WSOC showed some decrease in winter due to inactive biogenic emissions and reduced open biomass burning.

Relative contribution of WSOC and WIOC from different sources (i.e., fossil WIOC, BB/BG WIOC, fossil WSOC, and BB/BG WSOC) to total OC was also assessed for different seasons and two PM_2.5_ mass concentration regimes (i.e., PM_2.5_ >100 μg m^−3^ and PM_2.5_ <100 μg m^−3^) (Fig. 2C–E). In winter, under both regimes, fossil WIOC constituted the majority (51–55%) of OC mass, while BB/BG WSOC constituted the least (12–14% of OC). However, in summer days with PM_2.5_ >100 μg m^−3^, OC originated mainly from BB/BG WSOC (~41%), followed by BB/BG WIOC (~25%), fossil WSOC (~22%), and fossil WIOC (~12%). The high BB/BG contributions in high PM_2.5_ summer days might be attributed to burning of field crop residues during this period as well as SOA formation by photochemical oxidation of abundant biogenic volatile organic compounds (BVOCs)^27^,^48^.

**Figure 3:**
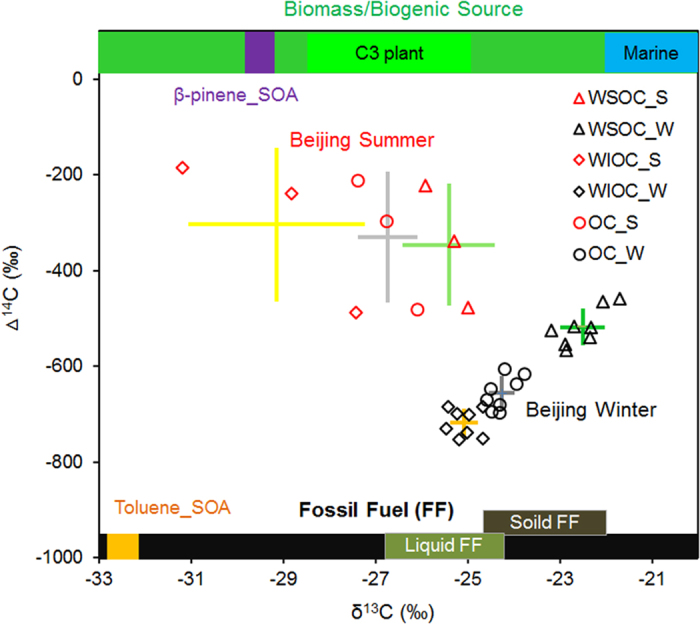
Two-dimensional dual-isotope δ^13^C-Δ^14^C signatures in WSOC (triangle), WIOC (diamond) and OC (circle) in PM_2.5_ samples collected during the summer (red) and winter (black) campaign in Beijing. Centers of cross bars indicate mean of all measured samples and length of bars represent one standard deviation. δ^13^C signatures for primary sources (e.g., C3 plant, Marine, Solid FF, liquid FF, where “FF” denotes “fossil fuel”) and secondary sources (Toluene_SOA and β-pinene_SOA) marked in this plot are based on reported literature values (see Table S2). Colors for the crosses are corresponding to the different shapes of symbols, presenting different types of OC. Yellow, gray and green crosses represent WIOC, OC and WSOC, respectively, and darker and lighter color indicates winter and summer samples, respectively.

Stable carbon isotope ratio (δ^13^C) can serve as a useful tracer to complement Δ^14^C in distinguishing origins of carbonaceous matter. The average δ^13^C for OC in this study (winter: −24.3 ± 0.3‰; summer: −26.7 ± 0.7‰) was comparable to the values reported earlier^49^ (Table S1). WSOC always had an enriched δ^13^C value compared to WIOC, suggesting there were differences in emission sources and/or atmospheric processing for WSOC and WIOC. The end-member δ^13^C values from different primary and secondary sources were summarized in Table S2. Combination of δ^13^C and Δ^14^C data further indicated that the relative contributions of specific source to OC and its two sub-constituents were distinct in winter and summer (Fig. 3). OC, WSOC and WIOC in summer PM_2.5_ samples (with PM_2.5_ > 100 μg m^−3^) were more modern, with prominent influence of BB/BG emissions (especially from C3 plants) and biogenic SOA. By contrast, all wintertime carbonaceous components showed more fossil origins and relatively enriched δ^13^C values.

It is clear that all the δ^13^C of wintertime WSOC (−22.5 ± 0.5‰) were located in the δ^13^C range for solid fuel (e.g. coal) emissions, except two outlier values, which might be influenced by air mass transported through the Bohai Sea (see air mass trajectory for 15-Jan and the day after, 16-Jan, Fig. S4). This indicated that there might be influence from marine aerosol, but not for all the winter samples. Good correlations between both fossil and BB/BG WSOC and levoglucosan (r^2^ = 0.69 and 060, respectively) suggested the wintertime BB/BG WSOC was related to residential biomass burning, while fossil source WSOC was associated with coal combustion rather than other fossil sources such as vehicle emissions and natural gas, in that levoglucosan is not emitted from the latter, but more likely from coal combustion besides biomass burning^50^,^51^,^52^,^53^. Based on back trajectories, the receptor site in Beijing could be influenced by air masses transported from other surrounding areas (Fig. S4), where residential coal and biomass burning were significant (Fig. S5). Thus, the enrichment of ^13^C in WSOC in winter seemed to be also related to long-range transport^28^, or influence from marine sources^54^, but the possible causes are still speculative. While, the δ^13^C in WSOC in summer were more closely located in the overlapped range for C3 plant and liquid fossil fuel, with stronger BB/BG source contributions to samples impacted by biomass burning activities^27^ while comparable contributions from fossil and BB-BG sources on other days.

WIOC (δ^13^C: −25.1 ± 0.3‰), the most abundant component of OC, was more modern in summer with influence by both primary and secondary biogenic sources, while dominated by fossil source such as solid (e.g. coal) and liquid (e.g. diesel, gasoline or crude oils) fossil fuels (Fig. 3) in winter. Good correlation of EC with wintertime fossil WIOC (r^2^ = 0.85) and BB/BG WIOC (r^2^ = 0.85) demonstrated the significance of combustion sources. Stronger correlation of levoglucosan with both fossil and BB/BG WIOC (r^2^ = 0.97 and r^2^ = 0.92, respectively) (Fig. S6), further indicated the dominant combustion influences from coal combustion and domestic biomass burning, which was probably due to emissions in the rural area of Beijing where both coal and biomass were both substantially used in winter for house heating and cooking. Here, we examined two major constituents of WIOC (n-alkanes and PAHs). Homologues of *n*-alkanes (C_9_–C_37_) showed a carbon preference index (CPI) close to 1 (1.01–1.26, average as 1.16 ± 0.09), indicating predominant fossil contribution from coal and/or petroleum combustion^55^,^56^. Diagnostic ratios of individual PAH such as Benzo[a]pyrene/Benzo[ghi]perylene (0.95 ± 0.10, >0.6), Benzo[a]anthracene/(Benzo[a]antharacene+Chrysene) (0.42 ± 0.05, >0.35), Fluoranthene/(Fluoranthene+Pyrene) (0.56 ± 0.02, >0.5), and Indeno[1,2,3-cd]pyrene/(Indeno[1,2,3]-cd)pyrene+Benzo[ghi]perylene) (0.54 ± 0.01, >0.5), all provide supportive evidences for major role of coal combustion and biomass burning (Table S3 and Fig. S7). Taken together, coal combustion (e.g., residential coal combustion, coal-fired industry and power plant) played a vital role in both the high fossil WIOC and WSOC mass measured during winter in Beijing.

### Source of light-absorbing OA components

**Figure 4:**
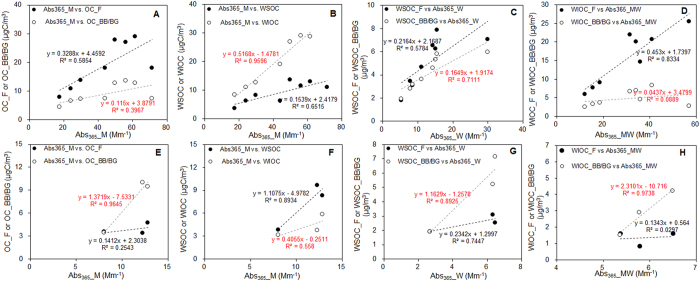
Scatter plots of relationship between Abs_365_ and both fossil and BB/BG source WSOC/WIOC/OC in winter (**A–D**) and Summer (**E–H**) (Note: “F” denotes “Fossil”, “BB/BG” denotes “Biomass burning/Biogenic”, “W” denotes “water extract”, “M” denotes “methanol extract”, “MW” denotes difference between methanol and water extracts).

To date, BB/BG has been treated as the most important source of BrC^9^, however, the ^13^C and ^14^C fingerprint here clearly demonstrate that fossil C could be a major contributor to OC during winter in Beijing. Total light absorption presented by Abs_365_ of methanol extracts (Abs_365__M) exhibited better correlation with WIOC and fossil OC in winter (Fig. 4A,B), and WSOC and BB/BG OC in summer (Fig. 4E,F). Wintertime Abs_365_ of water extracts (Abs_365__W) and Abs_365_ of water-insoluble extracts (Abs_365__MW, Abs_365_ of methanol extracts minus water extracts) correlated well with fossil WSOC and WIOC (e.g., Abs_365__W *vs.* fossil WSOC: r^2^ = 0.58; Abs_365__MW *vs*. fossil WIOC: r^2^ = 0.83), respectively (Fig. 4C,D), suggesting that the aforementioned high light-absorbing WS-BrC and WIS-BrC in Beijing winter was to a large extent attributed to fossil source emissions. It is noteworthy that wintertime Abs_365__W also correlated well with WSOC_BB/BG (r^2^ = 0.71), implying biomass burning was also an important source for wintertime WS-BrC. During summer, Abs_365__W correlated better with BB/BG WSOC (r^2^ = 0.89), and Abs_365__MW was more closely related to BB/BG WIOC (r^2^ = 0.97) (Fig. 4G,H). Taken together, correlation is better when one type of WSOC or WIOC from fossil or BB/BG dominates. That is, it is WSOC and BB/BG sources dominate in summer, while the dominant type in winter is WIOC and fossil sources.

### Significance of coal combustion

From the OC emission inventory, primary emissions from domestic/residential fossil fuel combustion (DOFU) and biomass burning (DOBI), and industry coal combustion (INCB) sector for central heating all increased in winter compared to summer in Beijing, with higher emissions from DOFU and INCB sectors compared to DOBI sectors (Fig. 1A and Fig. S3). Huang *et al*.^36^ indicated higher coal combustion contributions to organic aerosol than biomass burning and traffic emission during the same winter period. Zheng *et al*.^57^ revealed that low temperature residential coal combustion could explain 14% of the measured OC in Beijing in January, 2000. As higher emission factor and mass fraction of carbonaceous components in PM_2.5_ from lower temperature residential coal combustion were observed compared to industrial coal-fired boiler emissions, and carbonaceous aerosol from the former included more potential light-absorbers (e.g., PAHs, aromatic acids, phenolic compounds) and exhibited a stronger spectral dependence^58^,^59^, Chinese residential coal combustion was here for the first time investigated for its light-absorbing properties. The MAE_365_ of WSOC emitted from Chinese residential coal combustion averaged 1.10 ± 0.16 m^2^ g^−1^ C^−1^, comparable to or even slightly higher than MAE from biomass burning source testing in China^26^ (Table S4). MAE_365_ of WIOC from residential coal combustion was about 1.57 ± 0.78 m^2^ g^−1^ C^−1^, about 1.4-fold higher compared to the co-emitted WSOC and comparable to the ambient WIOC.

Although Beijing has recently started to switch from coal to cleaner and more efficient energy such as natural gas or liquefied petroleum gas, coal use was still more than 66% of China’s total primary energy consumption during 1978–2013 (Fig. S8). The impacts of coal combustion emission on air quality, optical properties and health impacts are of great concern in China. Substantial amount of coal use, along with massive low quality coal in use and low combustion efficiency is the key of this problem. Taken together, the high mass contribution of carbonaceous aerosol from fossil origin and its strong light-absorptivity suggests that light-absorption by fossil-origin OA should be considered in China, where significant amount of fossil fuel is still in use, especially as coal in the winter.

Conclusively, WSOC was the most prominent OA component in summer, yet exhibiting even higher mass absorption efficiency in winter with increased WSOC from solid fossil fuel (e.g. coal) combustion in Beijing. WIOC dominated the increased OC mass in winter, with an overwhelming contribution from fossil source especially coal combustion. WIOC also exhibited higher light-absorptivity in winter compared to summer, which could be stronger than that of WSOC. The combination of radiocarbon and stable carbon signatures, organic molecular tracers, and light-absorbing measurements here provides observational evidences that coal, besides biomass burning, can be a strong source of BrC, especially in regions where coal is still extensively used for heating and cooking during the heating season. Results from carefully-controlled source testing of Chinese-characteristic residential coal combustion in this study confirmed residential coal combustion could emit primary BrC with high light-absorptivity, but it can go through changes or aging process after emission. From the perspective of both coal source samples and wintertime ambient samples, fossil source BrC may play a crucial role in regional aerosol radiative forcing and climate. Fossil-originated BrC should be incorporated into climate- and chemical-transport models together with biogenic/biomass burning derived BrC. Furthermore, it is important to investigate detailed light-absorbing properties of brown carbon from different emission sources and its atmospheric processing including production by SOA formation and loss by photobleaching in the near future to improve our understanding of radiative forcing of the OA.

## Methods

### Ambient and source aerosol sampling

Ambient fine particulate matter (PM_2.5_) were collected on quartz filters by a high volume sampler (VFC-PM_2.5_, Thermo Fisher Scientific CO., U.S.) in January and June of 2013, on the roof of a building (20 m above the ground level) on the campus of Peking University (39°59′21″N, 116°18′25″E) located in the northwestern Beijing (Fig. S1). The sampling site is situated in the typical mixed districts of urban residential and commercial areas, representative of the typical Beijing urban area. Quartz filters (8 × 10 inch, 2500 QAT-UP, Pall Corp., NY, USA) for particle collection were prebaked at 550 °C for 6 h in a muffle furnace and wrapped in pre-combusted aluminum foil bags before sampling, and stored in triple plastic bags in a refrigerator under −20 °C after sampling until analysis. Samplers were operated at 1.13 m^3 ^min^−1^, and lasted for 23.5 hours for each sample. Field blanks were collected at the beginning and end of the sampling period.

Five kinds of coal combusted in different parts of China were provided by local residents, including coals mined in Shanxi province, Shandong province, Heilongjiang province and Inner Mongolia Municipality. Simulated laboratory burns of the coals were conducted in a traditional coal stove used in north China. The smoke was collected on quartz filters by a hood dilution sampling system, which was directly connected to the coal stove. Briefly, the system includes a hood, a fume channel, a dilution system, a residence and dilution chamber, as well as four PM_2.5_ cyclones.

### Chemical analysis

1.5 cm^2^ punches from each sample were taken and acidified by fumigation in an open glass petri dish held in a desiccator over 12 M hydrochloric acid for 24 h, and subsequently dried at 60 °C for 1 h to remove carbonates prior to analysis. OC and EC were measured using these fumigated subsamples with a thermal optical transmission analyzer (TOT) (Sunset Laboratory, Tigard, OR, USA) based on the NIOSH 5040 method. It should be noticed that the split time of OC and EC of each individual sample was recorded and noted during this analysis process.

Subsamples were extracted in Milli-Q water (≥18 MΩ) by ultra-sonication. An aliquot of the water extract of each sample was used for analyzing levoglucosan by high performance anion-exchange chromatography coupled with pulsed amperometric detection (HPAEC-PAD). And WSOC concentration was measured by a high-temperature catalytic instrument (Shimadzu-TOC-VCPH analyzer, Shimadzu, Kyoto, Japan) following the non-purgeable organic carbon (NPOC) protocol. A detailed description of this approach could be found in ref. 60. All WSOC results were blank corrected.

Source-diagnostic organic molecular components were analyzed by gas chromatography-mass spectrometry (GC-MS). Briefly, each filter sample was spiked with internal standards including Acenaphthene-d10, Anthracene-d10, Pyrene-d10, Benz[a]anthracene-d12, n-C15-d32, n-C20-d42, n-C24-d50, n-C30-d58, n-C32-d66, n-C36-d74, and ultrasonically extracted twice with 30 mL hexane/dichloride methane (DCM) (1:1, v/v) each time and then twice with 30 mL DCM/methanol (1:1, v/v) each time. Then the extracts were combined, filtered and concentrated to about 1 mL. Extracts were methylated with fresh prepared diazomethane to convert organic acids to methyl esters. The derivatized fractions were analyzed using an Aglient Model 5975 N gas chromatography-mass selective detector (GC-MSD) equipped with an HP-5 MS capillary column.

### Carbon isotope analysis

For the subsequent OC isolation step, 10 samples, including 7 samples in winter and 3 samples in summer were further selected for carbon isotope analysis. A detailed description of this approach could be found in previous studies^28^,^60^. Briefly, filter punches containing at least 100 μg OC were then acidified with the same method, and the TOT analyzer was slightly modified to enable cryogenic online user-defined isolation of the carbon dioxide (CO_2_) produced from the combustion of the OC portion of a sample. The resulting and separated CO_2_ was purified on-line through magnesium perchlorate and silver wool traps to remove water and halogen-containing gases, cryo-trapped in liquid nitrogen (N_2_) and sealed in glass ampoules containing silver (Ag, 2 or 3 grains) and copper oxide (CuO, about 50 μg). As for WSOC, the isolation of WSOC from atmospheric aerosol samples for ^13^C and ^14^C measurements followed the method in previous studies^30^,^37^, but with a slight difference in acidification procedure. Briefly, Milli-Q water extracts with at least 120 μg of WSOC were freeze-dried and then re-dissolved in 150 μl of 1 M hydrochloric acid to de-carbonate the samples before transferring into pre-combusted silver capsules, and finally evaporated and dried in an oven at 60 °C.

The OC-derived CO_2_ and WSOC isolates were then sent to U.S. National Ocean Sciences Accelerator Mass Spectrometry (NOSAMS) facility at Woods Hole Oceanographic Institution (MA, USA) for high-precision ^14^C-AMS analysis as described earlier^39^,^60^. The carbon content in the field blanks was negligible, being less than 0.4% of that measured in samples. Therefore, no field blank subtraction was performed for the isotope analysis in this study.

### Isotope-based source apportionment

Isotope measurement results were reported as fraction modern (*F*_*m*,_ proportionally to the ^14^C/^12^C-ratio), Δ^14^C-signature (the radiocarbon decay of a ^14^C/^12^C-standard that was established in the year 1950), as well as stable carbon isotope δ^13^C (^13^C/^12^C). A detailed description of the calculation of these parameters could be found in ref. 28. Fractional contributions of contemporary biomass/biogenic sources versus radiocarbon-extinct fossil fuel sources could be determined using the ^14^C isotopic mass balance equation:


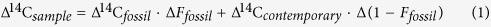


where 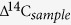 is the measured radiocarbon content of OC or WSOC from the sample, and 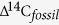 is −1000‰, since fossil carbon is completely depleted in radiocarbon. All ^14^C results are expressed as the fraction of modern carbon (*F*_m_) and have been corrected for their δ^13^C fraction, and further converted into the fraction of contemporary carbon (*F*_c_) by normalization with a conversion factor of 1.06^44^.

It should be noted that, concentrations and isotopic signatures of WIOC were derived from the WSOC and OC data based on an isotopic mass balance with following equations:


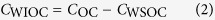



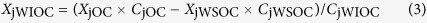


where variable *C* denotes “concentration” (μgC m^−3^); *X*_OC_, *X*_WSOC_ and *X*_WIOC_ represent the stable carbon or fraction modern (Fm) values for OC, WSOC and WIOC, respectively; *C*_jOC_, *C*_jWSOC_, *C*_jWIOC_ are the volume-normalized average concentrations of OC, WSOC, WIOC for the composite j.

### Light absorption measurement

Light absorption measurements were performed by a Shimadzu UV spectrophotometer (UV-1800) with scanning wavelength range of 340–1100 nm on aliquots of the Mill-Q water and methanol extracts, respectively. Methanol extracts were obtained with sample punches immersed in methanol for an hour without ultrasonic.

The light absorption coefficient Abs_λ_ at wavelength λ nm was calculated based on the directly obtained light attenuation data (ATN_λ_) by smoothing, subtracting reference interactions (at λ = 700 nm), and then completing conversion according to the formula:


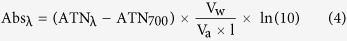


where ATN_λ_ is the light attenuation, V_w_ refers to the volume of water or methanol used for filter extraction, V_a_ is the volume of air passing through the filters when sampling, l is the length of the light absorbing path length (1 cm, for the currently used quartz cuvettes). In this study, λ= 365 nm was chosen due to its indication for light absorption of organic matter and avoiding interference from other inorganic species such as nitrate. The absorptivity per unit bulk material mass concentration (mass absorption efficiency, MAE) at 365 nm was calculated via the following equation:


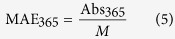


where *M* is the mass concentration of WSOC or WIOC. The wavelength dependence of the extracts absorption was investigated by fitting the absorption Angstrom exponent (AAE) (within the range of 340~400 nm) using the following relationship:


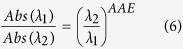


## Additional Information

**How to cite this article:** Yan, C. *et al*. Important fossil source contribution to brown carbon in Beijing during winter. *Sci. Rep.* **7**, 43182; doi: 10.1038/srep43182 (2017).

**Publisher's note:** Springer Nature remains neutral with regard to jurisdictional claims in published maps and institutional affiliations.

## Supplementary Material

Supplementary Materials

## Figures and Tables

**Table 1:** Source contributions and optical parameters of organic aerosol in ambient and source samples.

	Season	Organic Aerosol	Fraction in TOC^a^ (%)	*f*_Fossil (%)	Mass Absorption Efficiency^b^, MAE (m^2^ g^−1^ C^−1^)	Absorption Ångström Exponents^c^, AAE
Ambient	Winter	OC	1	67 ± 3%	−^d^	−^d^
		WSOC	31 ± 5%	54 ± 4%	1.43 ± 0.36	6.19 ± 0.43
		WIOC	69 ± 5%	73 ± 3%	1.46 ± 0.24^**e**^	6.00 ± 0.16
	Summer	OC	1	36 ± 13%	−^d^	−^d^
		WSOC	60 ± 6%	38 ± 12%	0.58 ± 0.14	6.31 ± 0.42
		WIOC	40 ± 6%	34 ± 15%	1.16 ± 0.38^**e**^	6.11 ± 0.37
Source testing	Coal^f^	WSOC	13 ± 10%	100%	1.10 ± 0.16	9.63 ± 1.78
		WIOC	87 ± 10%	100%	1.57 ± 0.78^e^	7.46 ± 0.77

Note: a. “TOC” refers to total organic carbon; b. is measured and calculated at 365 nm; c. is calculated at 340–400 nm; d. “−” means not measured or determined; “e” denotes values obtained with assumed methanol extract efficiency as 100%; f.. refers to residential coal combustion.
