# Supplementary material for: Important fossil source contribution to brown carbon in Beijing during winter
Source: Sci Rep. 2017 Mar 7;7:43182. doi: 10.1038/srep43182 (PMC5339816; doi:10.1038/srep43182)
Supplement: Supplementary Materials [file srep43182-s1.pdf]

Important fossil source contribution to brown carbon in Beijing during winter

Caiqing Yan[a], Mei Zheng[a,e*], Carme Bosch[b], August Andersson[b], Yury Desyaterik[c], Amy P. Sullivan[c], Jeffrey L. Collett, Jr.[c], Bin Zhao[d], Shuxiao Wang[d], Kebin He[d], Örjan Gustafsson[b*]

[a]State Key Joint Laboratory of Environmental Simulation and Pollution Control, College of Environmental Sciences and Engineering, Peking University, Beijing 100871, China

[b]Department of Environmental Science and Analytical Chemistry (ACES) and the Bolin Centre for Climate Research, Stockholm University, Stockholm 10691, Sweden

[c]Department of Atmospheric Science, Colorado State University, Fort Collins, Colorado 80523, USA

[d]State Key Joint Laboratory of Environment Simulation and Pollution Control, School of Environment, Tsinghua University, Beijing 100084, China

[e]The Beijing Innovation Center for Engineering Science and Advanced Technology, Peking University, Beijing 100871, China

***Corresponding authors:**

Mei Zheng
Tel: +86-10-62752436
Email: mzheng@pku.edu.cn

Örjan Gustafsson
Tel: +46 (0)70-3247317
Email: orjan.gustafsson@aces.su.se

Emission Inventory

Emission data in this study were supplied by Professor Shuxiao Wang's research group from Tsinghua University, which could be found in Wang et al. (1) and Zhao et al. (2, 3). And the method for developing yearly and monthly emission inventory was also described in some previous studies (1-4).

Bottom-up Emission Inventory *versus* Top-down ^{14}C Measurement

The approaches based on bottom-up emission inventory and top-town carbon isotope measurement both indicated that wintertime organic aerosol in Beijing was predominantly produced from fossil fuel combustion. The fossil fraction contribution (f-fossil) was slightly higher in the EI prediction (81%) relative to the ^{14}C-based observations (67±3%) in winter. However, there are some difference in results for summer. The reasons for the discrepancies between the EI and observation results could be attributed to (1) *Primary source vs. secondary source.* EI distinguishes contributions only from primary particulate OC, without considering the volatile organic compounds (VOCs) and associated secondary chemical transformation. However, ^{14}C method differentiates fossil and BB/BG origins of total OC including primary OC and secondary OC (SOC). Therefore, it is likely that the "overestimated" BB/BG fractions in winter by the observations compared to EI reflected the existence of biogenic SOA at the receptor site. Source apportionment results by Huang et al. (5) during the same study period quantified the biogenic SOA contribution to OA as 35-55%. Also, the secondary source contribution could be one of the reasons for the discrepancy occurred in summer. (2) *Anthropogenic source vs. biogenic source.* EI of OC here only includes particles emitted from anthropogenic sources, but not include primary biogenic emissions (such as plant wax). This might be the one of the most important reasons for the discrepancy in summer. (3) *Local emission vs. regional transport.* The ^{14}C results are based on measurement of ambient samples, which could include both local and transported particles, resulting in differences when comparing local EI results to the ^{14}C results. For examples, biomass burning in summer harvest season in other surrounding areas could also be transported to Beijing (6). (4) *Year of EI vs. observation*. Emission data was for the year of 2012 but ambient sampling was conducted in 2013, and (5) *Height of the emission sources.* Elevated point emission sources such as high stack of power plants are also included in the EI (Fig. S3), which might have smaller impacts compared to the ground sources. Despite the above factors, the bottom-up EI results and the top-down ^{14}C-based observations agreed reasonably well, especially in winter, the most severely polluted season in North China.

References

1. Wang SX, et al. (2014) Emission trends and mitigation options for air pollutants in East Asia. Atmos Chem Phys 14(13): 6571-6603.
2. Zhao B, et al. (2013a) Impact of national NO_x and SO_2 control policies on particulate matter pollution in China. Atmos Environ 77: 453-463.
3. Zhao B, et al. (2013b) Environmental effects of the recent emission changes in China: implications for particulate matter pollution and soil acidification. Environ Res Lett 8. DOI 10.1088/1748-9326/8/2/024031.
4. Fu X, et al. (2011) Emission inventory of primary pollutants and chemical speciation in 2010 for the Yangtze River Delta region, China. Atmos Environ 70: 39-50.
5. Huang RJ, et al. (2014) High secondary aerosol contribution to particualte pollution during haze events in China. Nature 514, 218-222.
6. Yan CQ, et al. (2015) Chemical characterisitcs and light-absorbing property of water-soluble organic carbon in Beijing: Biomass burning contributions. Atmos Environ 121: 4-12.

Figure and Table

Fig. S1. Map of emission inventory (EI) predictions of annual primary OC aerosol releases in China (Panel A), with spatially highly-resolved predictions for Beijing (BJ) in summer (Panel B) and winter (Panel C).

Fig. S2. Examples of light-absorption spectra of water and methanol extracts from samples collected in winter (W) and summer (S) in Beijing.

Fig. S3. Primary OC emission from different sectors in Beijing based on "bottom-up" emission inventory (Panel A) and ambient fossil and biomass/biogenic OC concentrations based on "top-down" ^{14}C observation (Panel B).

Fig. S4. 48-h backward air mass trajectories arrived at the sampling site in Beijing during winter study periods.

Fig. S5. Emission inventory of OC from different sectors in surrounding areas of Beijing (Data source: Tsinghua University, base year 2012).

Fig. S6. Correlation of fossil and BB/BG WSOC and WIOC with levoglucosan in winter.

Fig. S7. Cross-plots between diagnostic ratios for source identification of PAHs in wintertime Beijing.

Fig. S8. Energy consumption in China from 1980 to 2013. Panel A: Different type of energy consumptions; Panel B: Coal consumption by sectors

Table S1. Dual isotope ($\delta^{13}C$ and $\Delta^{14}C$) analysis of OC and its sub-fractions in ambient particles reported in different studies

Table S2. Range of $\delta^{13}C$ values from source sampling reported in previous studies

Table S3. Diagnostic ratios of PAHs compared with literature values

Table S4. MAE values of WSOC calculated at 365 nm for Chinese source samples

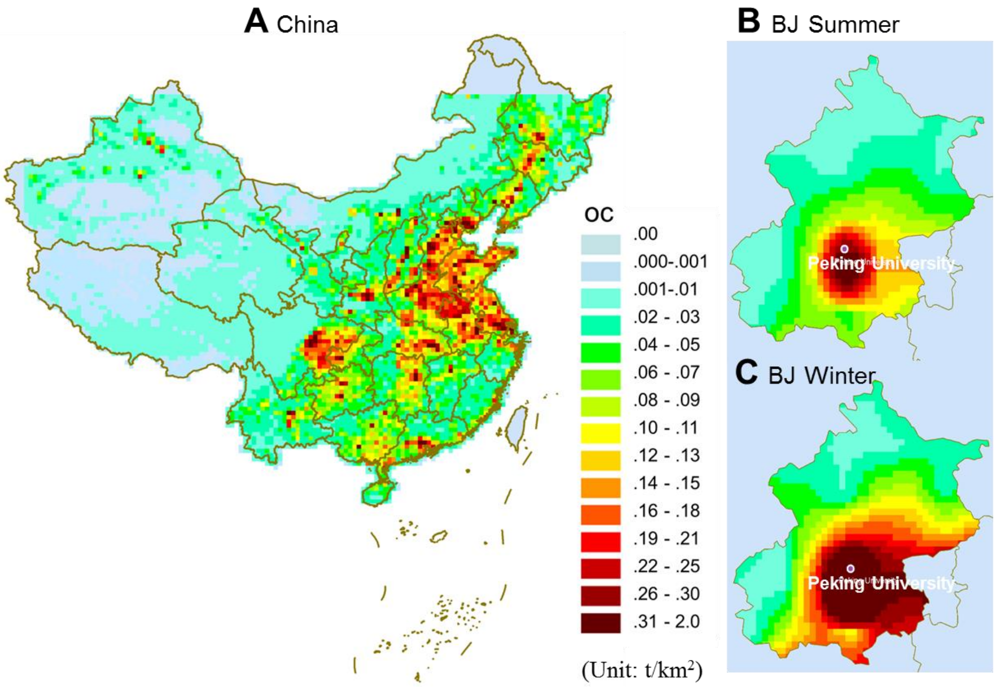

Fig. S1. Map of emission inventory (EI) predictions of annual primary OC aerosol releases in China (Panel A), with spatially highly-resolved predictions for Beijing (BJ) in summer (Panel B) and winter (Panel C). The maps were created using ArcGIS for Desktop (version 10.2.1, http://www.esri.com/software/arcgis/arcgis-for-desktop), with map layers from National Geomatics Center of China (http://ngcc.sbsm.gov.cn/). Anthropogenic OC emission data was supplied by coauthors from Tsinghua University (data source, see "Emission Inventory" in the supplementary).

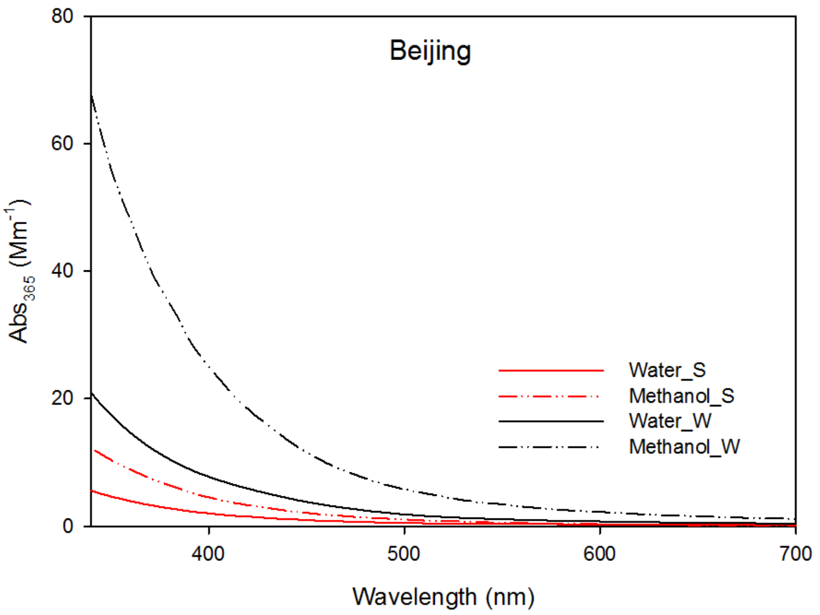

Fig. S2. Examples of light-absorption spectra of water and methanol extracts from samples collected in winter (W) and summer (S) in Beijing.

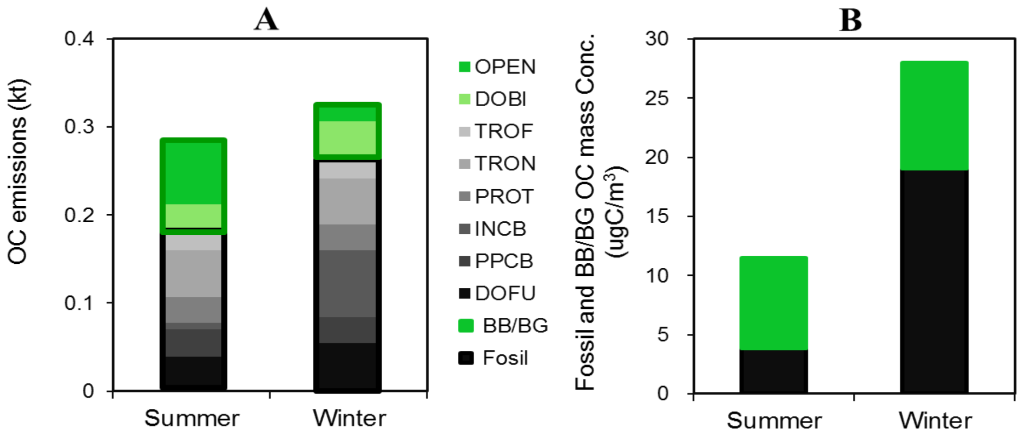

Fig. S3. Primary OC emission from different sectors in Beijing based on "bottom-up" emission inventory (Panel A) and ambient fossil and biomass/biogenic OC concentrations based on "top-down" ^{14}C observation (Panel B).

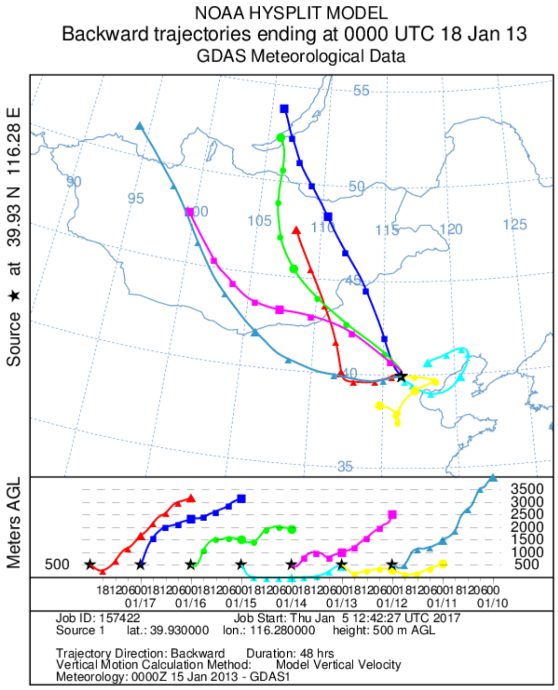

Fig. S4. 48-h backward air mass trajectories arrived at the sampling site in Beijing during 12-18 January, 2013. The image was created using Hybrid Single Particle Lagrangian Integrated Trajectory (HYSPLIT) model (version 4, Air Resources Laboratory, NOAA, USA, http://ready.arl.noaa.gov/HYSPLIT.php), with 48-h back trajectories for air masses at 500 m above ground level calculated. The authors gratefully acknowledge the NOAA Air Resources Laboratory (ARL) for the provision of the HYSPLIT transport and dispersion model and/or READY website (http://www.ready.noaa.gov) used in this publication.

Reference:

1. Stein, A.F., Draxler, R.R, Rolph, G.D., Stunder, B.J.B., Cohen, M.D., and Ngan, F., (2015). NOAA's HYSPLIT atmospheric transport and dispersion modeling system, Bull. Amer. Meteor. Soc., 96, 2059-2077.
2. Rolph, G.D. (2017). Real-time Environmental Applications and Display sYstem (READY) Website (http://www.ready.noaa.gov). NOAA Air Resources Laboratory, College Park, MD.

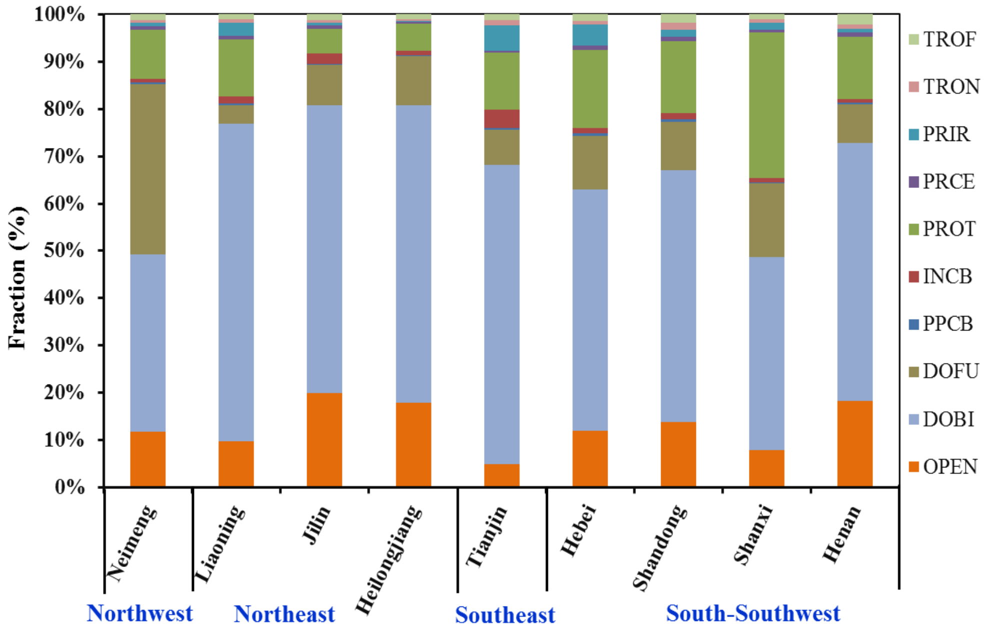

Fig. S5. Emission inventory of annual primary OC aerosol releases from different sectors in surrounding areas of Beijing (Data source: Tsinghua University, base year 2012)

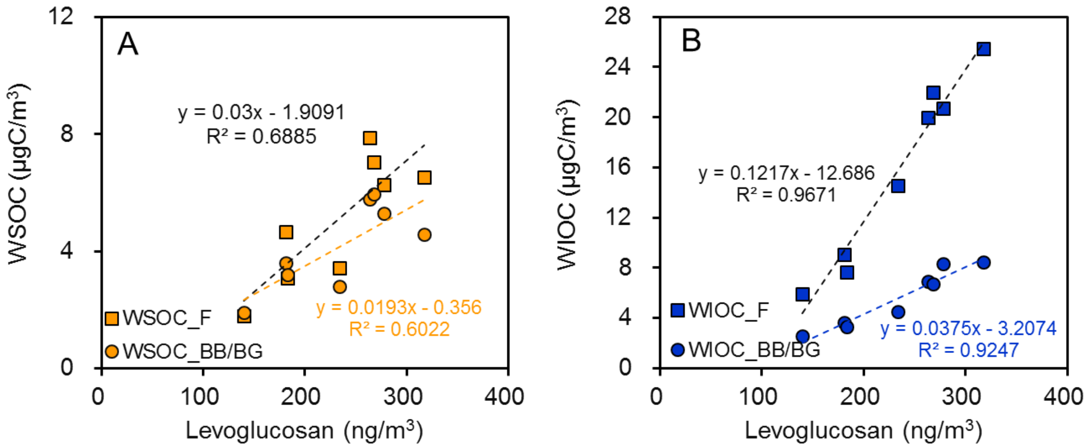

Fig. S6. Correlation of fossil and BB/BG WSOC and WIOC with levoglucosan in winter

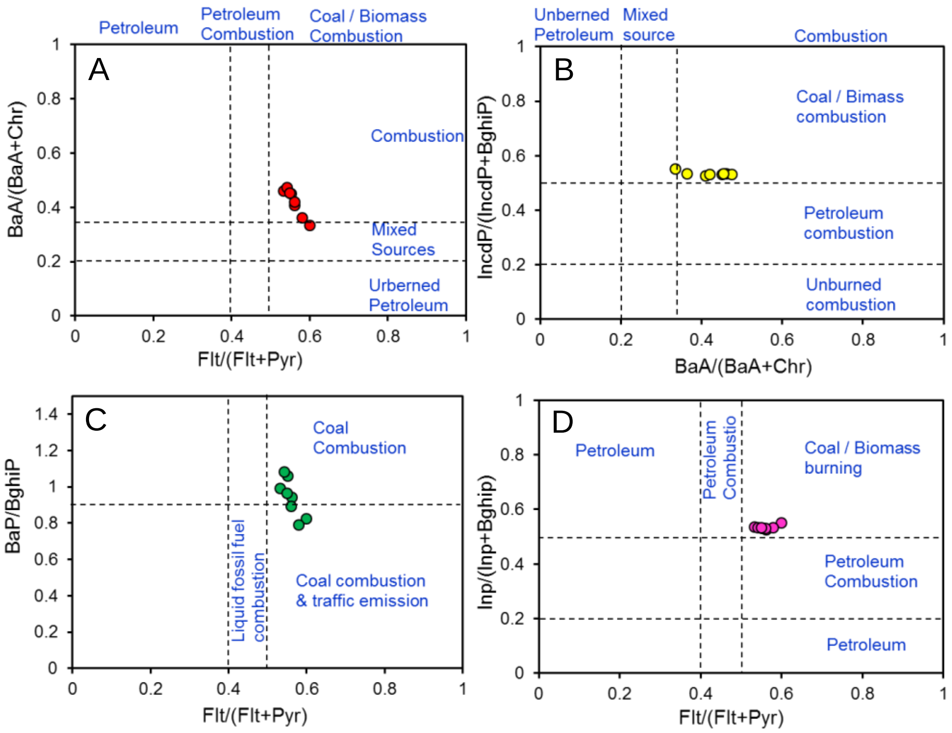

Fig. S7. Cross-plots between diagnostic ratios for source identification of PAHs in wintertime Beijing (Note: "BaA", "Chr", "IncdP", "BghiP", "BaP", "Flt", "Pyr" are short for "Benzo[a]anthracene", "Chrysene", "Indeno[1,2,3-cd]pyrene/(Indeno[1,2,3]-cd)pyrene", "Benzo[ghi]perylene", "Benzo[a]pyrene", "Fluoranthene", and "Pyrene", respectively).

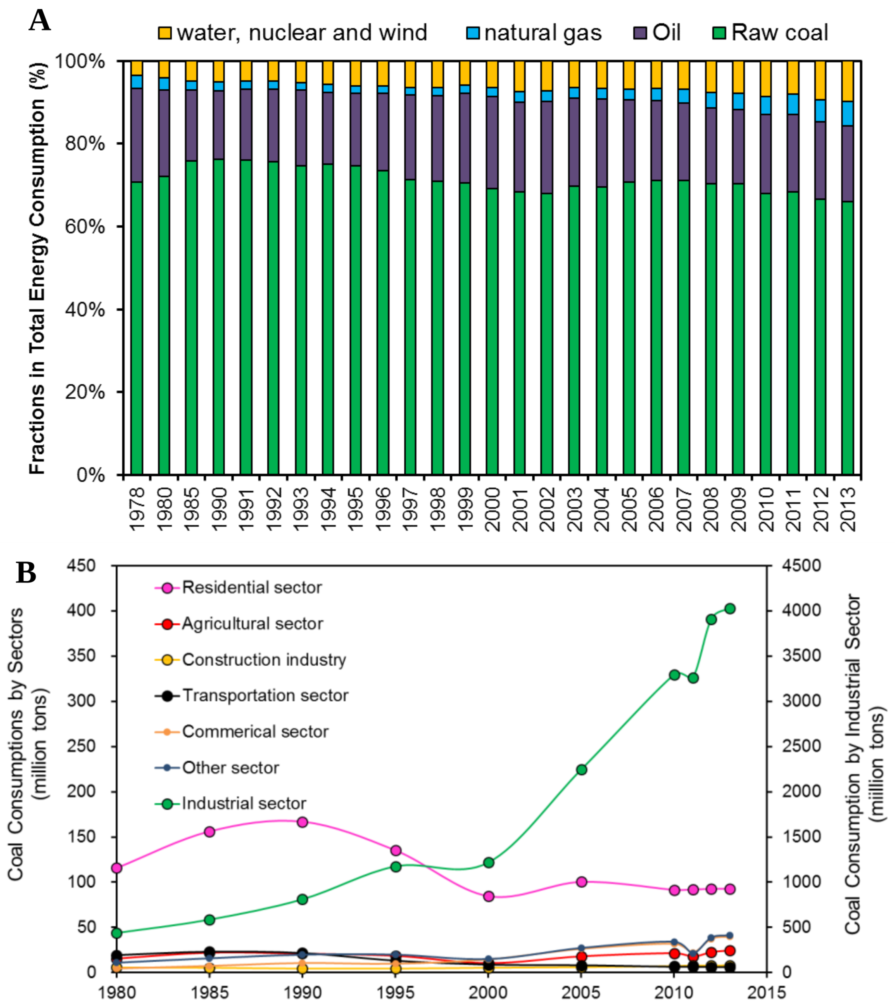

Fig. S8 Energy consumption in China from 1980 to 2013. Panel A: Different type of energy consumptions; Panel B: Coal consumption by sectors. (Data source: China Statistical Yearbook 2015 by National Bureau of Statistics).

Table S1. Dual isotope ($\delta^{13}C$ and $\Delta^{14}C$) analysis of OC and its sub-fractions in ambient particles reported in different studies

Sites	Season	Species	$\Delta^{14}C$	Fossil source	Non-fossil fraction	$\delta^{13}C$ value	Reference
SINH, India	Annual	WSOC	-80±40‰	23±4%		-20.4±0.5‰	(1)
MCOH, India	Annual	WSOC	-149±30‰	17±4%		-18.4±0.5‰	
MCOH	Winter	WSOC			86±5%	-20.8±0.7‰	(2)
		OC				-25.3±0.7‰	
New Delhi	Winter	WSOC	-118±40‰	21±4%		-24.1±1.0‰	(3)
		OC		46±8%			
KCOG	Spring	WSOC		26-49%			(4)
14 Chinese cities	Winter	OC				-26.62‰ to -23.08‰	(5)
	Summer	OC				-26.9‰ to -25.29‰	
Beijing	Winter	OC		58±5%	48±18%		(6)
Beijing	Winter	OC	-656.14±35.2	67.3±3.3%	32.7±3.3%	-24.26±0.29‰	This study
	Winter	WSOC	-518.66±38.5	54.2±3.7%	45.8±3.7%	-22.51±0.49‰	
	Winter	WIOC	-717.9±29.1	73.8±3.8%	26.1±3.8%	-25.08±0.31‰	
	Summer	OC	-330.77±137.42	36.4±23.1%	63.6±23.1%	-26.74±0.65‰	This study
	Summer	WSOC	-346.34±12.04	37.9±12.1%	62.1±12.1%	-25.40±0.46‰	
	Summer	WIOC	-304.14±160.87	34.0±15%	66.0±15%	-29.14±1.90‰	
Beijing	Winter	OC			38±3%		(7)

		WSOC		45 ±5%		
		WIOC		49 ±1%		
Shanghai	Winter	OC	49 ±2%	53 ±4% (biomass burning)		(6)
Xi’an	Winter	OC	38 ±3%	40 ±8%		(6)
Guangzhou	Winter	OC	35 ±7%	65 ±26% (biomass burning)		(6)
Guangzhou	Winter	WIOC, WSOC	40 ±6% 33 ±3%			(8)
		SOC	33 ±11%			
Guangzhou	Winter	OC		57 ±3%		(7)
		WSOC		71 ±5%		
		WIOC		45 ±4%		
Hong Kong	winter	OC			-26.9±0.6‰	(9)
Hainan Island	Fall	OC	19 ±10%	56 ±16% (biogenic) 44 ±14% (biomass burning)		(10)
		WSOC	17 ±10%			
Waliguan	Autuman to Spring	WIRC		67.2 ±7.7%	-25.3±0.8‰	(11)

Note: “MCOH” denotes Maldives Climate Observatory, Hanimaadhoo island; “SINH” denotes “Sinhagad in Indian”; “KCOG” denotes “Korea Climate Observatory at Gosan”; and “WIRC” denotes “Water-insoluble refractory carbon”

Reference

3. Kirillova EN, et al. (2013) ^{13}C- and ^{14}C-based study of sources and atmospheric processing of water-soluble organic carbon (WSOC) in South Asian aerosols. J Geophy Res 118(2): 614-626.
4. Bosch C, et al. (2014) Source-diagnostic dual-isotope composition and optical properties of water-soluble organic carbon and elemental carbon in the South Asian outflow intercepted over the Indian Ocean. J Geophys Res 119(20): 11743-711759.
5. Kirillova E.N, et al. (2014) Water-soluble organic carbon aerosols during a full New Delhi winter: Isotope-based source apportionment and optical properties. J Geophy Res 119, doi:10.1002/2013JD020041.
6. Kirillova EN, Andersson A, Han J, Lee M and Gustafsson Ö (2014) Sources and light absorption of water-soluble organic carbon aerosols in the outflow from northern China. Atmos Chem Phys 14(3): 1413-1422.
7. Cao JJ, et al. (2011) Stable carbon isotopes in aerosols from Chineses cities: Influence of fossil fuels. Atmos Enviorn 45: 1359-1363.
8. Zhang YL, et al. (2015) Fossil vs. non-fossil sources of fine carbonaceous aerosols in four Chinese cities during the extreme winter haze episode of 2013. Atmos Chem Phys 15: 1299-1312.
9. Zhang YL, et al. (2014) Micro-scale (ug) radiocarbon analysis of water-soluble organic carbon in aerosol samples. Atmos Enviorn 97: 1-5.
10. Liu JW, et al. (2014) Source apportionment using radiocarbon and organic tracers for $PM_{2.5}$ carbonaceous aerosols in Guangzhou, South China: contrasting local- and regional-scale haze events. Environ Sci Tech 48: 12002-12011.
11. Ho KF, et al. (2006) Variability of organic and elemental carbon, water soluble organic carbon, and isotopes in Hong Kong. Atmos Chem Phys 6: 4569-4576.
12. Zhang YL, et al. (2014) Radiocarbon-based source apportionment of carbonaceous aerosols at a regional background site on Hainan Island, South China. Environ Sci Technol 48: 2651-2659.
13. Zheng XD, Shen CD, Wan GJ, Tang J, Liu KX (2015) Mass and isotopic concentrations of water-insoluble refractory carbon in total suspended particulates at Mt. Waliguan Observatory (China). Particuology 20: 24-31.

Table S2. Range of $\delta^{13}C$ values from source sampling reported in previous studies

Sources	$\delta^{13}C$ value	Reference
C4 (corn, sugar cane, grass)	-18‰ to -8‰	(1,2)
C3 (wood)	-32‰ to -24.49 ‰ (-26.7±1.8‰)	(1-3)
Marine	-22‰ to -18‰	(4)
Solid fuel source (coal)	-24.9‰ to -21 ‰ (-23.38 ± 1.3‰)	
Liquid fuel	-28‰ to -24‰ (-25.5±1.3‰)	
Diesel	-28.4‰ to -24.9‰	(1, 5-12)
Gasoline	-25.8‰ to -23.9‰	
Vehicle	-27.5‰to -23.7‰	
Gas	-40‰ to -28‰	
β-pinene SOA	-29.6±0.2‰	(13)
Toluene-SOA	-32.5±0.3‰	(14)

Reference

1. Cai WW (2011) Study of stable carbon isotopic compositions of black carbon and individual PAHs from Typical Emission sources. Chinese Academy of Sciences, Yantai, China.
2. Smith BN, Epstein S (1971) Two categories of $^{13}C/^{12}C$ ratios for Higher Plants. Plant Physiol 47(3): 380-384.
3. Ancelet T, Davy PK, Trompetter WJ, Markwitz A, Weatherburn DC (2013) Carbonaceous aerosols in a wood burning community in rural New Zealand. Atmos Poll Res 4: 245-249.
4. Miyazaki Y, Kawamura K, Jung J, Furutani H, Uematsu M (2011) Latitudianl distributions of organic nitrogen and organic carbon in marine aerosols over the western North Pacific. Atmos Chem Phys 11: 3037-3049.
5. Ancelet T, Davy PK, Trompetter WJ, Markwitz A, Weatherburn DC (2011) Carbonaceous aerosols in an urban tunnel. Atmos Environ 45: 4463-4469.
6. Andersson A, et al. (2015) Regionally-varying combustion sources of the Janary 2013 severe haze events over eastern China. Environ Sci Tech 49: 2038-2043.
7. Agnihotri R, et al. (2011) Stable carbon and nitrogen isotopic composition of bulk aerosols over India and northern Indian Ocean. Atmos Environ 45: 2828-2835.
8. Chen YJ, Cai WW, Huang GP, Li J, Zhang G (2012) Stable Carbon Isotope of Black Carbon from Typical Emission Sources in China. Environ Sci 33: 673-678 (in Chinese) .
9. Gleason JD and Kyser TK (1984) Stable isotope compositions of gases and vegetation near naturally burning coal. Nature 307: 254-257.
10. Kawashima H, Haneishi Y (2012) Effects of combustion emsisions from the Eurasian continent in winter on seasonal delta C-13 of elemental carbon in aerosols in Japan. Atmos Environ 46: 568-579.
11. Tang GJ. (2001) $\delta^{13}C$ characterisitics of carboniferous coal in North China and its palaeogeographic implications. Political Scholars Anthology of Peking University. Peking University, Beijing, China..
12. Widory D (2006) Combustibles, fuels and their combustion products: A view through carbon isotopes, combust. Theory Modell 10: 831-841.
13. Fisseha R, et al. (2009) Stable carbon isotope composition of secondary organic aerosol from beta-pinene oxidation. J Geophys Res 114, D02304, doi:10.1029/2008JD011326.
14. Irei S, et al. (2006) Flow reactor studies of the stabel carbon isotope composition of secondary particulate organic matter generated by OH-radical-induced reactions of toluene. Atmos Environ 40: 5858-5867.

Table S3. Diagnostic ratios of PAHs compared with literature values

Ratios	This study	Values in Literature	Sources	Reference
IncdP/(IncdP+BghiP)	0.54±0.01	<0.2	Petrogenic	(1, 2)
		0.2-0.5	Petroleum Combustion	
		>0.5	Coal/Biomass Burning	
		0.56	Coal Combustion	
Flt/(Flt+Pyr)	0.56±0.02	<0.4	Petrogenic/Unburned Petroluem	(1, 3, 4)
		0.4-0.5	Liquid Fossil Fuel	(5)
		>0.5	Coal/Biomass Burning	
Ant/(Ant+Phe)	0.16±0.03	<0.1	Petrogenic	(4, 6)
		>0.1	Pyrogenic	
BaA/(BaA+Chr)	0.42±0.05	<0.2	Petrogenic	(1, 4, 7)
		0.2-0.35	Petrogenic and Coal Combustion	
		>0.35	Coal/Biomass Burning	
BaP/BghiP	0.95±0.10	<0.6	Traffic Emission	(8-10)
		>0.6	Non-traffic Fuel Combustion	(11)
		0.3-0.44	Gasoline/Diesel Engine	
		0.9-6.6	Coal combustion	

Reference

1. Yunker MB, et al. (2002) PAHs in the Fraser River basin: a critical appraisal of PAH ratios as indicators of PAH source and composition. Org Geochem 33: 489-515.
2. Tobiszewski M, Namieśnik J (2012) PAH diagnostic ratios for the identification of pollution eimssion sources. Environl Pollu 162: 110-119.
3. De La Torre-Roche RJ, Lee WY, Campos-Díaz SI (2009) Soil-borne polycyclic aromatic hydrocarbons in El Paso, Texas: analysis of a potential problem in the United

States/Mexico border region. J Hazard Mater 163: 946-958.
4. Hwang HM, Wade TL, Sericano JL (2003) Concentrations and source characterization of polycyclic aromatic hydrocarbons in pine needles from Korea,Mexico, and United States. Atmos Environ 37: 2259-2267.
5. Fadzil MF, Norhayati MT, Wan MK and Wan MZ (2008) Concentration and distribution of polycyclic aromatic hydrocarbons (PAHs) in the town of Kota Bharu, Kelantan Darul Naim. Malaysian J Anal Sci 12 (3): 609-618.
6. Pies C, et al. (2008) Characterization and source identification of polycyclic aromatic hydrocarbons (PAHs) in river bank soils. Chemosphere 72: 1594-1601.
7. Akyüz M, Çabuk H (2010). Gaseparticle partitioning and seasonal variation of polycyclic aromatic hydrocarbons in the atmosphere of Zonguldak, Turkey. Sci Total Environ 408: 5550-5558.
8. Pandey PK, Patel KS, Lenicek J (1999) Polycyclic aromatic hydrocarbons: need for assessment of health risks in India? Study of an urban-industrial location in India. Environ Monit Assess 59: 287–319.
9. Zhang XL, et al. (2005) Source diagnostics of polycyclic aromatic hydrocarbons based on species ratios: a multimedia approach. Environ Sci and Tech 39: 9109-9114.
10. Park SS, Kim YJ, Kang CH (2002) Atmospheric polycyclic aromatic hydrocarbons in Seoul, Korea. Atmos Environ 36: 2917–2924
11. Kavouras LG, Lawrence J, Koutrakis P, Stephanou EG, Oyola P (1999) Measurement of particulate aliphatic and polynuclear aromatic hydrocarbons in Santiago de Chile source reconciliation and evaluation of smapling artifacts. Atmos Environ 33: 4977-4986.

Table S4. MAE values of WSOC calculated at 365 nm for Chinese source samples

Source	Types	MAE_{365} ($m^2 g^{-1} C^{-1}$)	Std.[a]	Reference
Biomass	Wood	0.97	0.26	Du et al., 2014
	Grass	0.9	0.07	
	Crop straw	1.05	0.08	
Fossil source	Tractor	1.33	0.49	
	Motorcycle	0.2	0.08	
	Coal	1.10	0.16	This study

Note: “a” denotes one standard deviation.

Reference

1. Du Z, et al. (2014) A yearlong study of water-soluble organic carbon in Beijing I: Sources and its primary vs. secondary nature. Atmos Environ 92: 514-521.
